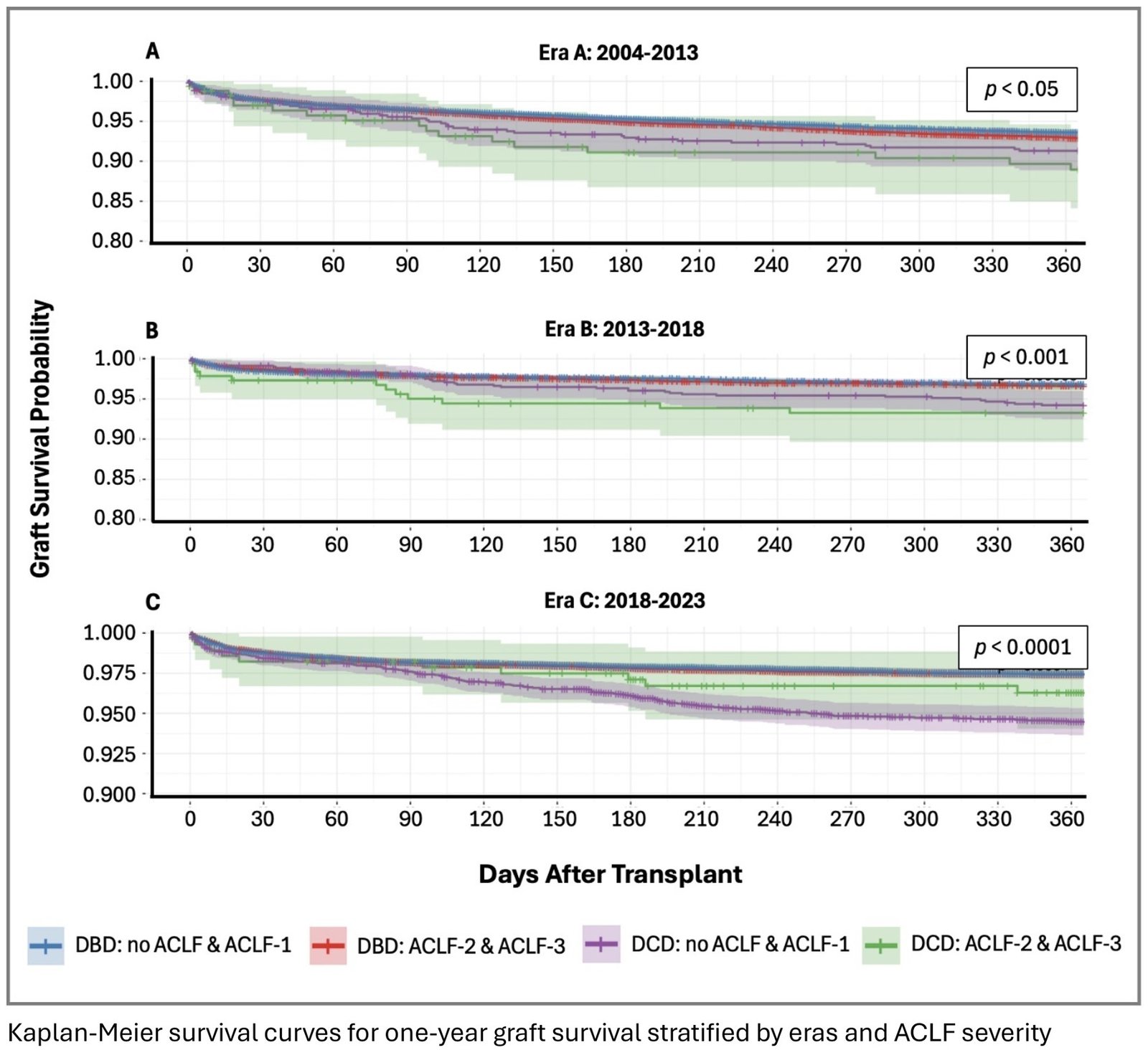# Poster Session I - A185 EVOLVING OUTCOMES AFTER LIVER TRANSPLANTATION (LT) FOR ACUTE-ON-CHRONIC LIVER FAILURE (ACLF): AN ERA ANALYSIS OF DONATION AFTER CIRCULATORY DEATH (DCD) VS DONATION AFTER BRAIN DEATH (DBD) GRAFTS

**DOI:** 10.1093/jcag/gwaf042.185

**Published:** 2026-02-13

**Authors:** Y Yao, T Wen, N W Tjandra, K Lau, Y Lin, X Chen, D Chahal

**Affiliations:** Department of Internal Medicine, University of British Columbia, Vancouver, BC, Canada; Department of Gastroenterology & Hepatology, University of British Columbia, Vancouver, BC, Canada; Department of Gastroenterology & Hepatology, University of British Columbia, Vancouver, BC, Canada; Department of Gastroenterology & Hepatology, University of British Columbia, Vancouver, BC, Canada; Department of Gastroenterology & Hepatology, University of British Columbia, Vancouver, BC, Canada; Department of Gastroenterology & Hepatology, University of British Columbia, Vancouver, BC, Canada; Department of Gastroenterology & Hepatology, University of British Columbia, Vancouver, BC, Canada

## Abstract

**Background:**

Research comparing DCD and DBD graft use in ACLF recipients is limited. While DCD grafts help expand the donor pool, they have historically been associated with higher risks of dysfunction and graft loss compared with DBD grafts. However, advances in allocation policy, donor management, and perioperative care may have altered these outcomes over time.

**Aims:**

To evaluate temporal trends in one-year graft survival (GS) and graft failure (GF) for DCD versus DBD grafts in ACLF patients.

**Methods:**

Using the Scientific Registry of Transplant Recipients (SRTR), adult LT recipients were divided into three eras: A (2004–2013), B (2013–2018), C (2018–2023). ACLF severity was classified per EASL-CLIF as non-critical (no ACLF/ACLF-1) or critical (ACLF-2/3). Exclusion criteria included prior LT, fulminant liver failure (status 1A), hepatocellular carcinoma, and multi-visceral transplantation (except liver–kidney). GS and GF were analyzed using Kaplan–Meier methods with log-rank tests and Cox proportional hazards models.

**Results:**

67,201 LT recipients were included (Era A: 25,075 DBD, 694 DCD; Era B: 13,997 DBD, 884 DCD; Era C: 25,809 DBD, 3,219 DCD). Among critical recipients, DCD grafts had significantly higher GF risk than DBD grafts in eras A (HR 1.88, *p*=0.01) and B (HR 2.28, *p*=0.006), but not in C (HR 1.60, *p*=0.14). Non-critical DCD grafts had higher GF risk than critical DBD grafts in all eras. For DBD grafts, non-critical recipients had lower GF risk than critical recipients in Era A (HR 0.85, *p*=0.002), but this difference was not significant in later eras. GS for critical DCD recipients improved across eras (A: 88.9%, B: 93.3%, C: 96.3%). In earlier eras, critical DCD recipients had the lowest GS (Era A: 88.9%, p < 0.05; Era B: 93.3%, p < 0.001). In Era C, non-critical DCD recipients had the lowest GS (94.5%, p < 0.0001).

**Conclusions:**

While DCD grafts were associated with inferior one-year survival across all eras, outcomes for critically ill ACLF-2/3 recipients improved over time. In the most recent era, no significant survival difference was observed between DCD and DBD grafts for critically ill ACLF recipients, supporting the safe expansion of DCD use in high-risk ACLF patients.

A185 Table 1: Multivariable stratified Cox proportional hazards regression model for one-year graft failure by era

The reference category was DBD with ACLF-2 and 3. Abbreviations: DBD, donation after brain death; DCD, donation after circulatory death; ACLF, acute-­on-chronic liver failure; HR, hazard ratio; CI, confidence interval

**Funding Agencies:**

CIHR, NoneDr. Daljeet Chahal receives salary or grant support from: Michael Smith Foundation for Health Research (MSFHR) Vancouver Coastal Health Research Institute (VCHRI) Canadian Institutes of Health Research (CIHR) Canadian Donation and Transplantation Research Program (CDTRP) Organ Donation and Transplant Research Foundation of BC